# CD147 as a novel biomarker for predicting the prognosis and clinicopathological features of bladder cancer: a meta-analysis

**DOI:** 10.18632/oncotarget.19257

**Published:** 2017-07-15

**Authors:** Hongru Li, Yadong Xu, Hui Li

**Affiliations:** ^1^ Xiangya Hospital, Central South University, Changsha, Hunan Province, China; ^2^ Reproductive Department, Xiangya Hospital, Central South University, Changsha, Hunan Province, China

**Keywords:** bladder cancer, CD147, prognosis, clinicopathological features, meta-analysis

## Abstract

**Objective:**

To assess the prognostic and clinicopathological characteristics of CD147 in human bladder cancer.

**Methods:**

Studies on CD147 expression in bladder cancer were retrieved from PubMed, EMBASE, the Cochrane Library, Web of Science, China National Knowledge Infrastructure, and the WanFang databases. Outcomes were pooled with meta-analyzing softwares RevMan 5.3 and STATA 14.0.

**Results:**

Twenty-four studies with 25 datasets demonstrated that CD147 expression was higher in bladder cancer than in non-cancer tissues (OR=43.64, P<0.00001). Moreover, this increase was associated with more advanced clinical stages (OR=73.89, P<0.0001), deeper invasion (OR=3.22, P<0.00001), lower histological differentiation (OR=4.54, P=0.0005), poorer overall survival (univariate analysis, HR=2.63, P<0.00001; multivariate analysis, HR=1.86, P=0.00036), disease specific survival (univariate analysis, HR=1.65, P=0.002), disease recurrence-free survival (univariate analysis, HR=2.78, P=0.001; multivariate analysis, HR=5.51, P=0.017), rate of recurrence (OR=1.91, P=0.0006), invasive depth (pT2∼T4 vs. pTa∼T1; OR=3.22, P<0.00001), and histological differentiation (low versus moderate-to-high; OR=4.54, P=0.0005). No difference was found among disease specific survival in multivariate analysis (P=0.067), lymph node metastasis (P=0.12), and sex (P=0.15).

**Conclusion:**

CD147 could be a biomarker for early diagnosis, treatment, and prognosis of bladder cancer.

## INTRODUCTION

Bladder cancer is the ninth most common cancer worldwide [1]. It was estimated that 76,960 new cases (4.6% of all new cancer cases) and 16,390 deaths would occur due to bladder cancer in the United States in 2016 [2]. High-grade invasive bladder cancer usually has a worse prognosis than low-grade superficial tumors. Unfortunately, approximately 30% newly diagnosed bladder cancer patients have invasive cancer [3], and 10-20% of superficial tumors will progress to invasive diseases. Thus, prediction of the progression of bladder cancer in certain patients remains a major challenge. Invasive cystoscopy is the most common method to detect suspected bladder cancer, because of the low sensitivity of other diagnostic tests, like urine cytology [3]. Bladder cancer has different biological characteristics, and patients with the same disease stage may have divergent clinical courses and different outcomes after receiving the same therapy [4]. Therefore, there is an urgent need for more sensitive and specific biomarkers that provide reliable information for tumor diagnosis, and elucidate the biological behavior of these tumors. This may allow more precise assessment of and better-targeted effective therapy for bladder cancer. A number of biomarkers such as survivin [5], fascin [6], MCT1, MCT4, and CD147 [7] were found to be involved in the development and progression of bladder cancer. Among these biomarkers, CD147, or extracellular matrix metalloproteinase inducer (EMMPRIN), is a transmembrane protein that acts as an important mediator of tumor cell invasion [8]. It is overexpressed in various tumors such as breast, lung, oral, esophageal, laryngeal, and renal cancers [9–14]. *In vitro* suppression of CD147 has been shown to inhibit the proliferation, migration, and invasion of T24 bladder cancer cells [15]. There are also a number of studies on CD147 expression profiles in bladder cancer patients, which indicate that CD147 may serve as a prognostic biomarker. However, some of these study results are controversial. For example, some studies showed that the positive expression of CD147 predicts poor overall survival (OS) [4, 7, 15-18], while Afonso et al. [16] and Choi et al. [7] declared no significance of positive or negative expression of CD147. Furthermore, Afonso et al. [16] and Han et al. [19] claimed that CD147 predicts poor disease specific survival (DSS), which contradicts the results of Hemdan et al. [17]. Some studies showed a significant association between CD147 over-expression and tumor stage [7, 20], while others disputed this [21]. The same conflict was also seen in the studies of lymphatic invasion status [7, 22], TNM stage [21, 23], and recurrence status [18, 24]. Therefore, we conducted this meta-analysis to quantitatively evaluate the relationship between CD147 and clinicopathological features and survival of bladder cancer patients.

## RESULTS

### Literature search and characteristics of included studies

**Figure 1:**
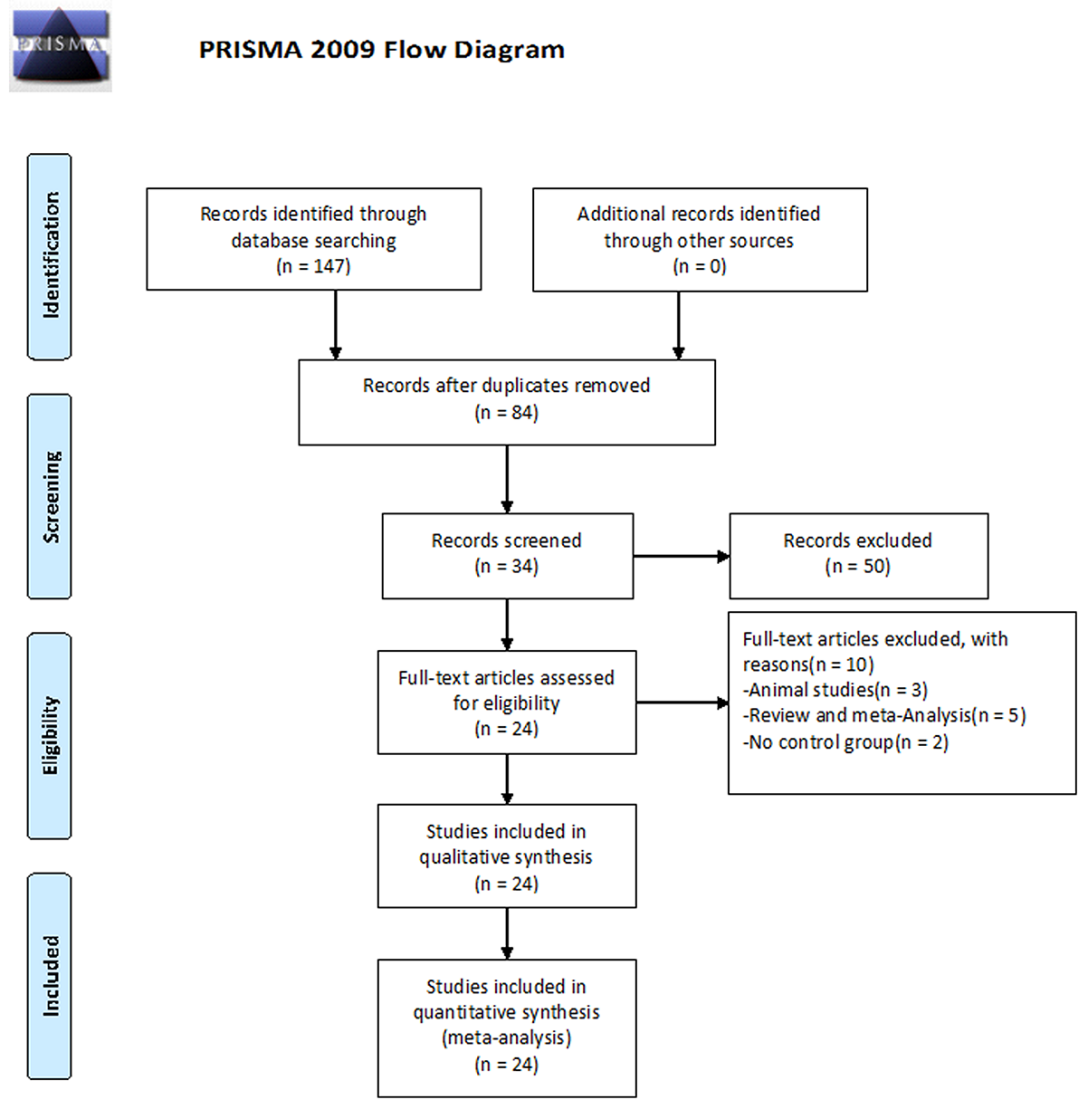
Flowchart of selection of studies for inclusion in meta-analysis A total of 147 studies were identified, and 63 studies were excluded because of duplication. After reading the titles and abstracts, 50 studies were excluded. 34 possible full text studies were carefully reviewed (animal studies [n = 3]; review and meta-analysis [n = 5]; no control group [n = 2]). Finally, 24 studies were included for quantitative analysis

A total of 147 studies were identified, of which 63 were excluded because of duplication. After reading the titles and abstracts, a further 50 studies were excluded. The remaining 34 full text studies were carefully reviewed (animal studies [n=3]; review and meta-analysis [n=5]; no control group [n=2]). Finally, 24 studies [4, 7, 15-36] were included for quantitative analysis (Figure 1).

The characteristics of these studies are summarized in Table 1. A total of 2493 patients, with or without bladder cancer, are included in 24 studies mostly in China (sixteen studies conducted in China [15, 18, 19, 21-25, 28-35], one from Portugal [16], one from Sweden [17], one from Korea [7], one from Egypt [20], one from India [26], one from Germany [27], one from Denmark [4], and one from Japan [36]). 8 studies [4, 7, 15-19, 22] with 9 datasets (the study by Hemdan et al. [17] had 2 datasets, grouped based on differences in treatment) are included in the survival analysis (Table 2). The pathological types of bladder cancer were transitional cell carcinoma, squamous cell carcinoma, and adenocarcinoma. The studies were published between 1993 and 2015. Immunohistochemistry (IHC) was used to detect the expression of CD147 in all studies, while reverse transcriptase polymerase chain reaction, Western blot, array analysis, immunoelectron microscopy, and nucleic acid in situ hybridization were also used in six studies [4, 21, 22, 26, 27, 36].

**Table 1:** Characteristics of eligible studies.

First author	Year	Origin	Cases	Median age	Counting method	Definition of CD147 positive	NOS score
Afonso, J [16]	2015	Portugal	114	70	IHC (SABC)	A1+B1≥4	8
Hemdan, T [17]	2015	Sweden	250	-	IHC (LP)	-	7
Li, F [25]	2015	China	66	Case, 64.28 Control, 63.47	IHC (SP)	A≥26%	7
Choi, JW [7]	2014	Korea	360	69	IHC (EnVision)	A2+B2≥16	7
Min, L [22]	2014	China	128	Case, 58.1 Control, 55.3	IHC (SP)	A>0% or weak intensity	7
El-Rehim, DM [20]	2013	Egypt	125	56	IHC (SABC)	A3*B1>2	7
Bhagirath, D [26]	2012	India	60	Case, 58 Control (Benign Prostatic Hyperplasia), 50.3 Control (Healthy), 48	ELISA	-	8
Wittschieber, D [27]	2011	Germany	103	71	IHC (SP)	B1≥2	7
Xue, YJ [15]	2011	China	128	Case, 58.3	IHC (SP)	B>0	8
Gao, LJ [24]	2011	China	50	68	IHC (SABC)	A4+B3>0	7
Li, M [21]	2011	China	79	60.5	IHC (SP)	A≥25%	7
Han, ZD [19]	2010	China	58	56.8	IHC (ABC)	A≥6%	8
Zhong, WD [18]	2010	China	131	Case, 68.1	IHC (EnVision)	A>5%	8
Chen, QB [28]	2010	China	93	Case, 71.5	IHC (SP)	A5*B1>1	7
Cui, W [29]	2010	China	50	Case, 61.8	IHC (SP)	A>0%	7
He, HC [30]	2009	China	103	Case, 61.5	IHC (SP)	A5*B1>1	7
Peng, XH [23]	2009	China	64	Case, 59.9	IHC (SP)	A>0%	7
Lin, JX [31]	2008	China	84	Case, 61.5 Control, 56.9	IHC (SP)	A>0%	7
Als, AB [4]	2007	Denmark	124	-	IHC (SP)	A≥10%	8
Gao, L [32]	2007	China	59	Case, 57.8	IHC (SP)	A>25% or B>0	7
Li, M [33]	2007	China	84	Case, 61.5 Control, 56.9	IHC (SP)	A≥25%	7
Li, WL [34]	2007	China	68	62	IHC (SP)	A≥25% or B>0	7
Han, JL [35]	2003	China	54	61.6	IHC (SP)	A≥25% or B>0	7
Muraoka, K [36]	1993	Japan	58	-	IHC (ABC)	A>30%	7

Streptavidin-perosidase (SP), Labeled- perosidase (LP), Avidin-biotin complex (ABC), Streptavidin-avidin-biotin complex (SABC)

**A:Positive cell percentage**

A1: scored 0 (0 %), 1 (<5 %), 2 (5–50 %), 3 (>50 %).

A2: scored 0-10.

A3: scored 1 (<25 %), 2 (25–50%), 3 (51–75%), 4 (>75%).

A4: scored 0 (<10%), 1 (10-50 %), 2 (>50%).

A5: scored 0 (≤5%), 1 (6-25%), 2 (26-50%), 3 (≥50%)

**B: Staining intensity**

B1: scored 0 (absence of staining), 1 (weak staining), 2 (moderate staining), 3 (strong staining).

B2: scored 0-10.

B3: scored 0 (absence of staining), 1 (pale brown), 2 (dark brown)

**Table 2:** Characteristics of the included studies

First author	Time	Country	Cases	Tumor types	Test method	Cut-off value(positive)	Follow-up time(months)	Survival results	HR with 95%CI	Source of data
Afonso, J [16]	2015	Portugal	114	Bladder cancer	IHC (SABC)	A1+B≥4	1-132	OS, DFS	OS(U), 4.1(1.27,13.18) DFS(U), 2.98(1.13,7.84)	Curve + p-value
Hemdan, T [17]	2015	Sweden	250	Bladder cancer	IHC (LP)	-	-	OS, DSS	OS#(U), 3.11(1.19,8.09) OS*(U), 1.44(0.79,2.61) DSS#(U), 1.71(1.06,2.74) DSS*(U), 1.13(0.67,1.92) OS(M), 1.64(1.091,2.467) DSS(M), 1.428(0.876,2.328)	Curve + Direct
Choi, JW [7]	2014	Korea	360	Bladder cancer	IHC (EnVision)	A2+B2≥16	median, 36	OS	OS(U), 2.58(0.84,7.98)	Curve
Min, L [22]	2014	China	128	Bladder cancer	IHC (SP)	A>0% or weak intensity	11-86	DSS	DSS(U), 3.14(1.12,8.76) DSS(M), 3.035,(1.462,6.301)	Curve + Direct
Xue, YJ [15]	2011	China	86	Bladder cancer	IHC (SP)	B>0	3-86	OS	OS(U), 3.783(1.935,7.395) OS(M), 2.332(1.149,4.734)	Direct
Han, ZD [19]	2010	China	58	Bladder cancer	IHC (ABC)	A≥6%	12-60	DSS	DSS(U), 3.08(1.11,8.49)	Curve + p-value
Zhong, WD [18]	2010	China	131	Bladder cancer	IHC (EnVision)	A>5%	36	OS, DFS	OS(U), 2.42(1.29,4.54) DFS(U), 2.68(1.29,5.54) OS(M), 3.31(1.068,15.72) DFS(M), 5.11(1.052,17.23)	Curve + Direct
Als, AB [4]	2007	Denmark	124	Bladder cancer	IHC (SP)	A≥10%	15-60	OS	OS(U), 3.93(1.74,8.89)	Indirect

**U:** univariate analysis.

**M:** multivariate analysis.

A:Positive cell percentage

A1: scored 0 (0 %), 1 (<5 %), 2 (5–50 %), 3 (>50 %).

A2: scored 0-10.

A3: scored 1 (<25 %), 2 (25–50%), 3 (51–75%), 4 (>75%).

A4: scored 0 (<10%), 1 (10-50 %), 2 (>50%).

A5: scored 0 (≤5%), 1 (6-25%), 2 (26-50%), 3 (≥50%)

**B: Staining intensity**

B1: scored 0 (absence of staining), 1 (weak staining), 2 (moderate staining), 3 (strong staining).

B2: scored 0-10.

B3: scored 0 (absence of staining), 1 (pale brown), 2 (dark brown)

**#: chemotherapy prior to cystectomy**

*: cystectomy

### Qualitative assessment

The study quality was assessed using the Newcastle-Ottawa quality assessment scale (NOS), generating scores ranging from 7 to 8 (with a mean of 7.25), where a higher value (0-9) indicates better methodology. The results of the quality assessment are shown in Table 1 , with detailed information shown in Supplementary Table 1.

### CD147 expression and survival analysis

#### CD147 expression and OS

**Figure 2:**
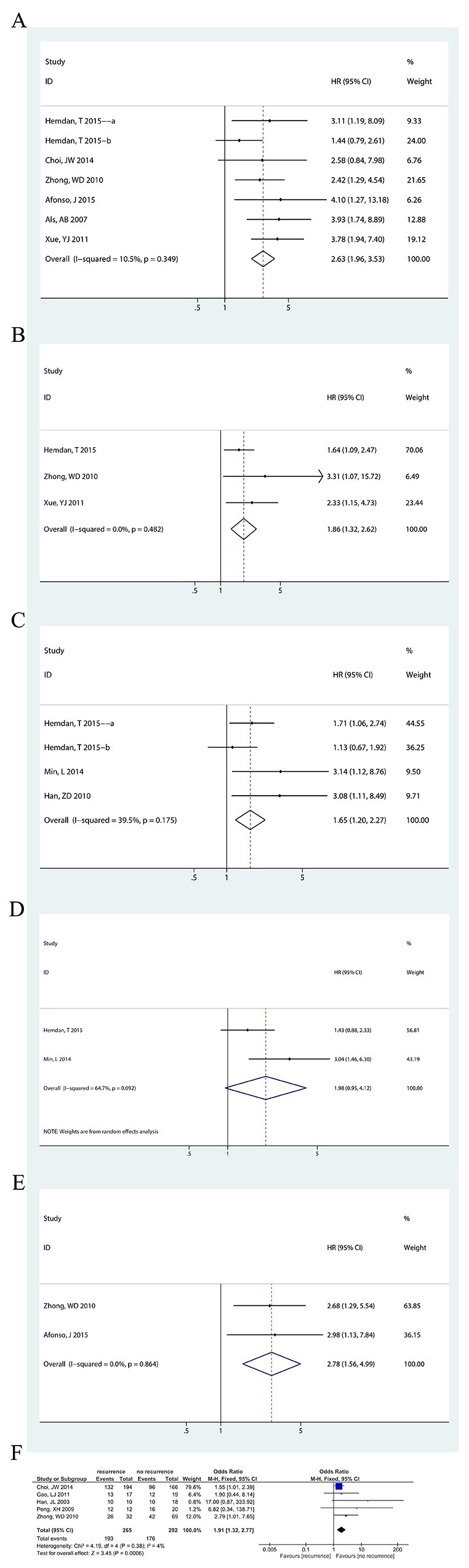
Forest plots of CD147 expression and survival analysis The squares and horizontal lines correspond to the study-specific OR and 95% CI. The area of the squares reflects the study-specific weight (inverse of the variance). The diamonds represent the pooled OR/HR and 95% CI. The solid vertical line is at the null value (OR/HR=1). **(A)** The relationship between CD147 expression and overall survival (for univariate data; Hedman, T 2015 a: data with neoadjuvant chemotherapy and cystectomy as the therapeutic regimen, Hemdan, T 2015 b: data with only cystectomy as the therapeutic regimen). CD147 expression was associated with overall survival (HR=2.63, 95%CI= [1.96, 3.53], P<0.00001). **(B)** The relationship between CD147 expression and overall survival (for multivariate data). CD147 expression was associated with overall survival (HR=1.86, 95%CI= [1.32, 2.62], P=0.00036). **(C)** The relationship between CD147 expression and disease specific survival (for univariate data; Hedman, T 2015 a: data with neoadjuvant chemotherapy and cystectomy as the therapeutic regimen, Hemdan, T 2015 b: data with only cystectomy as the therapeutic regimen). CD147 expression was associated with disease specific survival (HR=1.65, 95%CI= [1.20, 2.27], P=0.002). **(D)** The relationship between CD147 expression and disease specific survival (for multivariate data). CD147 expression was associated with disease specific survival (HR=1.98, 95%CI= [0.95, 4.12], P=0.067). **(E)** The relationship between CD147 expression and disease recurrence-free survival (for univariate data). CD147 expression was associated with disease specific survival (HR=2.78, 95%CI= [1.56, 4.99], P=0.001). **(F)** The relationship between CD147 expression and recurrence. CD147 expression was associated with recurrence (OR=1.91, 95%CI= [1.32, 2.77], P=0.0006).

A univariate analysis of OS was performed in 6 studies [4, 7, 15-18], including seven datasets. Without heterogeneity (P=0.349, I^2^=10.5%), fixed-effects model showed that the CD147-positive group had a lower OS (HR=2.63, 95% CI=[1.96, 3.53], P<0.00001). In addition, a multivariate analysis of OS was performed in 3 studies [15, 17, 18], without heterogeneity (P=0.482, I^2^=0%), and a fixed-effects model was used. The result was the same (HR=1.86, 95% CI=[1.32, 2.62], P=0.00036) (Figure 2A, 2B).

#### CD147 expression and DSS

Three studies [17, 19, 22], including four datasets, showed DSS with a univariate analysis. Without heterogeneity (P=0.175, I^2^=39.5%), fixed-effects model showed that the CD147-positive group had lower OS (HR=1.65, 95% CI=[1.20, 2.27], P=0.002). In addition, Hemdan et al. [17] and Min et al. [22] reported a multivariate analysis with a random-effects model, showing no difference between CD147-positive and -negative group survival (HR=1.98, 95% CI=[0.95, 4.12], P=0.067), with heterogeneity (P=0.092, I^2^=64.7%) (Figure 2C, 2D).

#### CD147 expression and DFS

Zhong et al. [18] and Afonso et al. [16] performed a univariate analysis for disease recurrence-free survival (DFS); however only Zhong et al. [18] performed a multivariate analysis, which included 101 patients, with a 3-year follow-up. Fixed-effects model showed that the CD147-positive group had poorer DFS survival (univariate analysis, P=0.864, I^2^=0%, HR=2.78, 95% CI=[1.56, 4.99], P=0.001; multivariate analysis, HR=5.51, 95% CI=[1.36, 22.32], P=0.017) (Figure 2E).

#### CD147 and recurrence

Five studies [7, 18, 23, 24, 35], utilizing 557 tissue samples, investigated the relationship of CD147 expression with the recurrence of bladder cancer. Without heterogeneity (P=0.38, I^2^=4%), the fixed-effects model showed a significant difference between the recurrence group and the no-recurrence group (OR=1.91, 95% CI=[1.32, 2.77], P=0.0006) (Figure 2F).

#### Subgroup analysis of survival data

We conducted a subgroup analysis based on IHC and cut-off values. This revealed that CD147 expression is correlated with a poor prognosis for bladder cancer in almost all subgroups (P<0.05), except multivariate analysis of OS (P=0.081) and DSS (P=0.068) in positive cell percentage only subgroup and multivariate analysis of DSS (P=0.153) in non- streptavidin-perosidase (SP) subgroup. This may attributes to the insufficiency of sample size and studies. In summary, our results are reliable (Table 3).

**Table 3:** Results of subgroup analysis of survival data

	Based on methods of IHC				Based on cut-off value			
	Subgroups (datasets)	HR&OR	95%CI	P value	Subgroups (datasets)	HR&OR	95%CI	P value
OS (U)	SP (2)	3.842	2.289-6.448	<0.001	Based on positive cell percentage only (1)	3.93	1.739-8.883	0.01
	Non-SP (5)	2.203	1.545-3.142	<0.001	Others (6)	2.480	1.812-3.394	<0.001
OS (M)	SP (1)	2.332	1.149-4.734	0.019	Based on positive cell percentage only (1)	3.310	0.863-12.699	0.081
	Non-SP (2)	1.740	1.178-2.571	0.005	Others (2)	1.790	1.257-2.550	0.001
DSS (U)	SP (1)	3.140	1.123-8.782	0.029	Based on positive cell percentage only (1)	3.080	1.114-8.518	0.030
	Non-SP (3)	1.543	1.106-2.153	0.011	Others (3)	1.544	1.106-2.155	0.011
DSS (M)	SP (1)	3.035	1.462-6.301	0.003	Based on positive cell percentage only (0)	-	-	-
	Non-SP (1)	1.428	0.876-2.328	0.153	Others (2)	1.979	0.951-4.114	0.068
DFS (U)	SP (0)	-	-	-	Based on positive cell percentage only (1)	2.68	1.29-5.54	0.008
	Non-SP (2)	2.785	1.556-4.985	0.001	Others (1)	2.98	1.13-7.84	0.027
Recurrence	SP (2)	11.08	1.35-90.77	0.02	Based on positive cell percentage only (2)	3.14	1.21-8.14	0.02
	Non-SP (3)	1.73	1.18-2.52	0.005	Others (3)	1.73	1.15-2.58	0.008

Overall survival (OS), Disease specific survival(DSS), Disease recurrence-free survival (DFS), univariate analysis (U), Streptavidin-perosidase (SP), multivariate analysis (M).

Non-SP includes labeled- perosidase (LP), avidin-biotin complex (ABC), streptavidin-avidin-biotin complex (SABC) and EnVision.

Others are based on staining intensity or both staining intensity and positive cell percentage.

OS (U), OS (M), DSS (U), DSS (M) and DFS (U) used HR as effect size and recurrence used OR as effect size.

### CD147 expression in different bladder tissues

#### CD147 in bladder cancer and non-cancerous tissues

**Figure 3:**
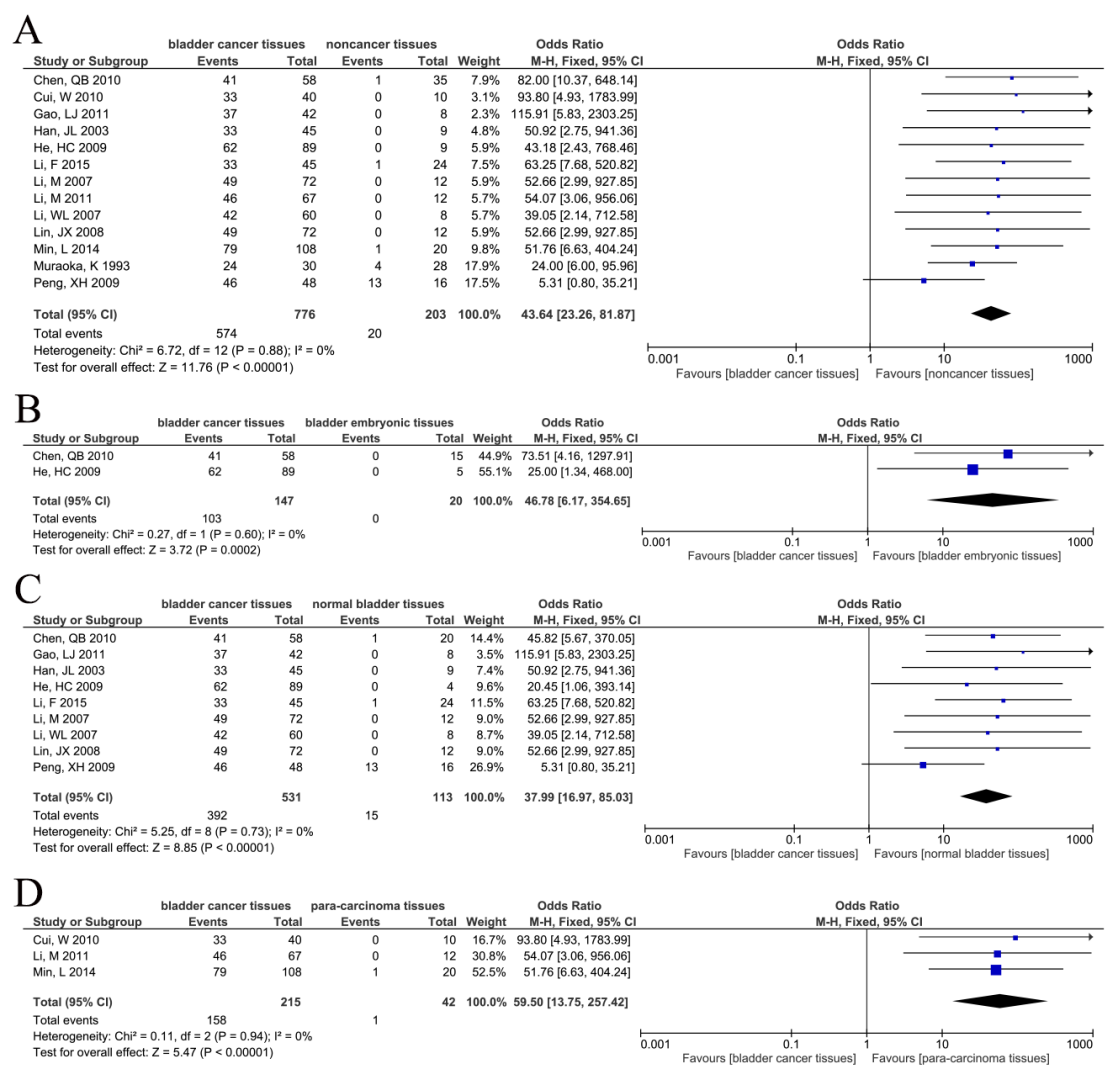
Forest plots of CD147 expression in different types of bladder tissues The squares and horizontal lines correspond to the study-specific OR and 95% CI. The area of the squares reflects the study-specific weight (inverse of the variance). The diamonds represent the pooled OR and 95% CI. The solid vertical line is at the null value (OR=1). **(A)** CD147 positive expression between bladder cancer tissues and non-cancer bladder tissues. Significant difference was found between two groups (OR=43.64, 95%CI=[23.26, 81.87], P<0.00001). **(B)** CD147 positive expression between bladder cancer tissues and bladder embryonic tissues. Significant difference was found between two groups (OR=46.78, 95%CI=[6.17, 354.65], P=0.0002). **(C)** CD147 positive expression between bladder cancer tissues and normal bladder tissues. Significant difference was found between two groups (OR=37.99, 95%CI=[16.97, 85.03], P<0.00001). **(D)** CD147 positive expression between bladder cancer tissues and para-carcinoma tissues. Significant difference was found between two groups (OR=59.50, 95%CI=[13.75, 257.42], P<0.00001).

The positive expression of CD147 in bladder cancer and non-cancerous tissues was investigated in 13 studies [21-25, 28-31, 33-36] with 979 patients. Without heterogeneity (P=0.88, I^2^=0%), the fixed-effects model showed that CD147 expression was higher in bladder cancer tissues (OR=43.64, 95% CI=[23.26, 81.87], P<0.00001) (Figure 3A).

#### CD147 expression in bladder cancer and bladder embryonic tissues

Two studies [28, 30] reported the positive expression of CD147 in bladder cancer tissues and bladder embryonic tissues, including 147 bladder cancer tissues and 20 bladder embryonic tissues. Without heterogeneity (P=0.603, I^2^=0%), the fixed-effects model showed that positive expression of CD147 in bladder cancer tissues was higher than in bladder embryonic tissues (OR=46.78, 95% CI=[6.17, 354.65], P=0.0002) (Figure 3B).

#### CD147 expression in bladder cancer and normal bladder tissues

Nine studies [23-25, 28, 30, 31, 33-35] investigated the positive expression of CD147 in bladder cancer tissues and normal bladder tissues, including 531 bladder cancer tissues and 113 normal bladder tissues. Fixed-effects model showed that CD147 positive expression was greater in bladder cancer tissues (OR=37.99, 95% CI=[16.97, 85.03], P<0.00001), without heterogeneity (P=0.73, I^2^=0%) (Figure 3C).

#### CD147 in bladder cancer and para-carcinoma tissues

Three studies [21, 22, 29] reported the expression of CD147 in bladder cancer tissues and para-carcinoma tissues, including 215 bladder cancer tissues and 42 para-carcinoma tissues. Fixed-effects model showed a higher rate of CD147 expression in the bladder cancer group (OR=59.50, 95% CI=[13.75, 257.42], P<0.00001), without heterogeneity (P=0.94, I^2^ =0%) (Figure 3D).

### Correlation of CD147 expression with clinicopathological parameters

#### CD147 and clinical stage

**Figure 4:**
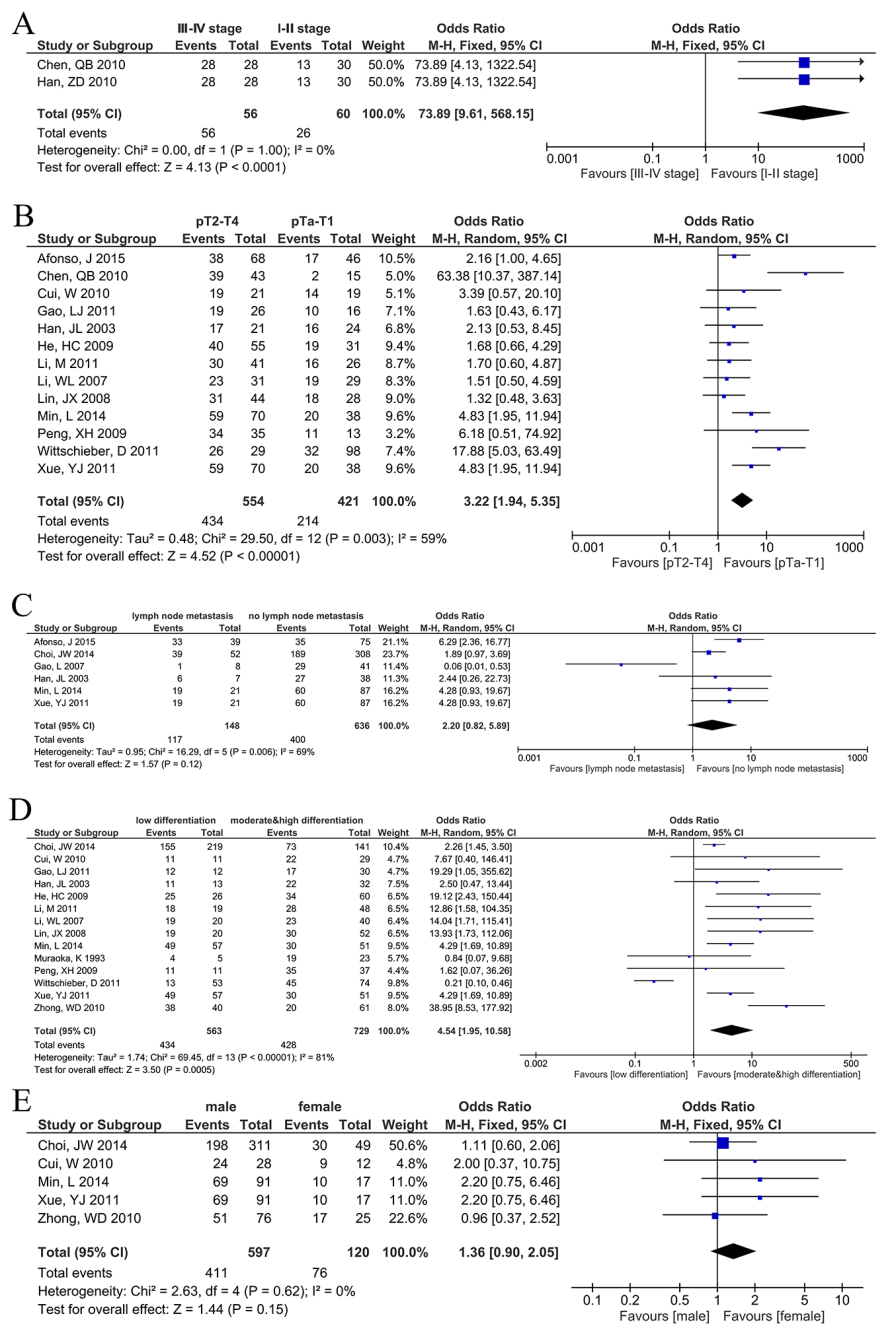
Forest plots of CD147 expression and clinicopathological features of bladder cancer patients The squares and horizontal lines correspond to the study-specific OR and 95% CI. The area of the squares reflects the study-specific weight (inverse of the variance). The diamonds represent the pooled OR and 95% CI. The solid vertical line is at the null value (OR=1). **(A)** The relationship between CD147 expression and clinical stage. Significant difference was found between TNM III-IV stage and TNM I-II stage (OR=73.89, 95%CI=[9.61, 568.15], P<0.0001). **(B)** The relationship between CD147 expression and invasive depth. Significant difference was found between pT2-T4 group and pTa-T1 group (OR=3.22, 95%CI=[1.94, 5.35], P<0.00001). **(C)** The relationship between CD147 expression and lymph node metastasis. CD147 expression wasn’t associated with lymph node metastasis (OR=2.20, 95%CI=[0.82, 5.89], P=0.12). **(D)** The relationship between CD147 expression and histological differentiation. CD147 positive expression was associated with low differentiation (OR=4.54, 95%CI=[1.95, 10.58], P=0.0005). **(E)** The relationship between CD147 expression and gender. CD147 expression wasn’t associated with gender (OR=1.36, 95%CI=[0.90, 2.05], P=0.15).

Clinical stage is an international standard for tumor staging. Stages are grouped into two categories, with TNM I-II having a better prognosis than TNM III-IV. The association between CD147 and clinical stage was investigated in two studies [19, 28], which showed that CD147 expression in the TNM III-IV stage group was greater than that in TNM I-II stage group, with fixed-effects model (OR=73.89, 95% CI=[9.61, 568.15], P<0.0001) and without heterogeneity (P=1.00, I^2^=0%) (Figure 4A).

#### Correlation of CD147 with invasive depth

Invasive depth also named tumor stage, from pTa to pT4, indicates deeper infiltration of the bladder wall. The association between CD147 and invasive depth was investigated in 13 studies [15, 16, 21-24, 27-31, 34, 35]. With significant heterogeneity (P=0.003, I^2^=59%), the random-effects model showed a significant difference between the pT2-T4 group and the pTa-T1 group (OR=3.22, 95% CI=[1.94, 5.35], P<0.00001) (Figure 4B).

#### CD147 expression and lymph node metastasis

Six studies [7, 15, 16, 22, 32, 35] reported the relationship between CD147 expression and lymph node metastasis of bladder cancer. Heterogeneity was observed in the analysis (P=0.006, I^2^=69%), and therefore, a random-effects model was used. The results showed no association between CD147 expression and lymph node metastasis (OR=2.20, 95% CI=[0.82, 5.89], P=0.12) (Figure 4C).

#### CD147 and histological differentiation

Histologically, bladder cancer is categorized into low, moderate, and high differentiations. Fourteen studies [7, 15, 18, 21-24, 27, 29-31, 34-36] investigated the association between CD147 expression and histological differentiation. With significant heterogeneity (P<0.00001, I^2^=81%), the results showed that CD147 expression was associated with low differentiation (OR=4.54, 95% CI=[1.95, 10.58], P=0.0005) (Figure 4D).

#### CD147 and sex

Five studies [7, 15, 18, 22, 29] involving 717 patients, reported the relationship between CD147 expression and sex. Fixed-effects model showed no difference between male and female groups (OR=1.36, 95% CI=[0.90, 2.05], P=0.15), with no heterogeneity (P=0.62, I^2^=0%) (Figure 4E).

### Sensitivity analysis and publication bias

**Figure 5:**
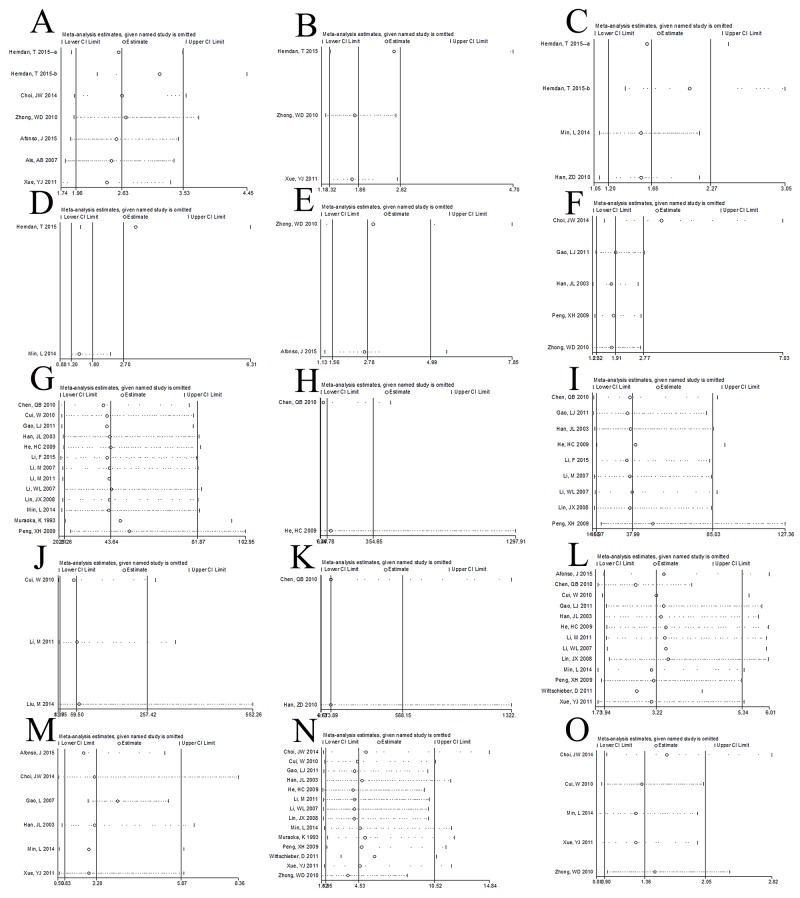
Sensitivity analysis We exclude study one by one to evaluate the influences of individual studies on the final effect and all the results were consist with the result of including all studies, which means our results are stable and reliable. **(A)** CD147 expression and overall survival with univariate analysis; **(B)** CD147 expression and overall survival with multivariate analysis; **(C)** CD147 expression and disease specific survival with univariate analysis; **(D)** CD147 expression and disease specific survival with multivariate analysis; **(E)** CD147 expression and disease recurrence-free survival with univariate analysis; **(F)** CD147 expression and recurrence; **(G)** CD147 positive expression between bladder cancer tissues and non-cancer bladder tissues; **(H)** CD147 positive expression between bladder cancer tissues and bladder embryonic tissues; **(I)** CD147 positive expression between bladder cancer tissues and normal bladder tissues; **(J)** CD147 positive expression between bladder cancer tissues and para-carcinoma tissues; **(K)** CD147 expression and clinical stage; **(L)** CD147 expression and invasive depth; **(M)** CD147 expression and lymph node metastasis; **(N)** CD147 expression and histological differentiation; **(O)** CD147 expression and gender.

**Figure 6:**
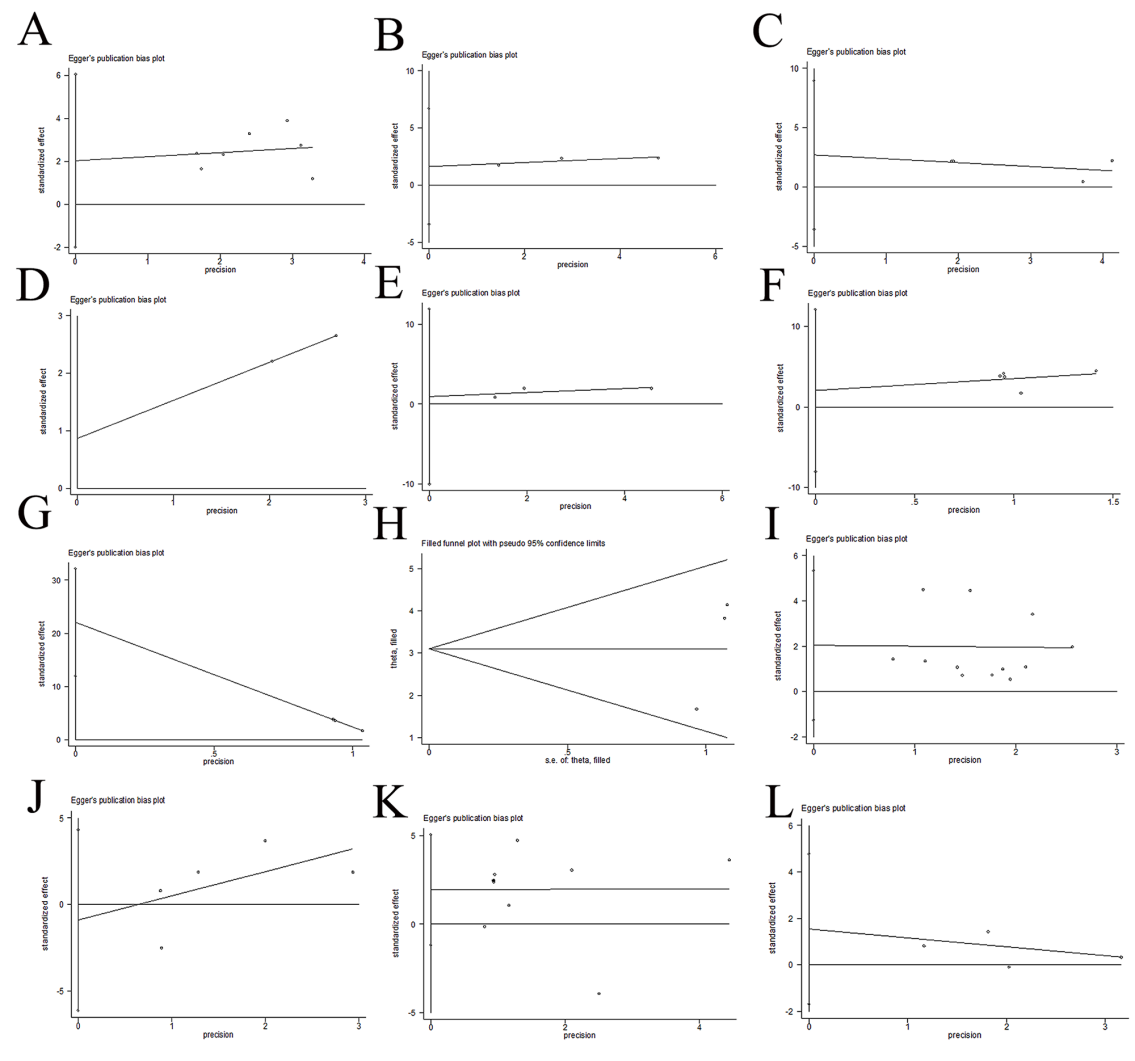
Publication bias No publication bias was found by egger’s test except for CD147 positive expression between bladder cancer tissues and normal bladder tissues. **(A)** CD147 expression and overall survival with univariate analysis; **(B)** CD147 expression and overall survival with multivariate analysis; **(C)** CD147 expression and disease specific survival with univariate analysis; **(D)** CD147 expression and disease specific survival with multivariate analysis; **(E)** CD147 expression and recurrence; **(F)** CD147 positive expression between bladder cancer tissues and non-cancer bladder tissues; **(G)** CD147 positive expression between bladder cancer tissues and normal bladder tissues; **(H)** CD147 positive expression between bladder cancer tissues and normal bladder tissues (trim and fill method); **(I)** CD147 expression and invasive depth; **(J)** CD147 expression and lymph node metastasis; **(K)**: CD147 expression and histological differentiation; **(L)** CD147 expression and gender.

A sensitivity analysis was performed to evaluate the reliability of the results. While multiple datasets were available, sensitivity was tested by excluding studies one by one. Except in the case of Lymph node metastasis vs. No lymph node metastasis, all analyses were stable. To test for publication bias, we use Egger's test. All P values were higher than 0.05, indicating no publication bias. In summary, the results were stable and reliable (Table 4, Figure 5 and Figure 6).

**Table 4:** Summary of sensitivity and publication bias analysis.

	OR/HR fluctuation	95%CI/HR fluctuation	Publication bias (P value)
**CD147 expression and survival analysis**			
OS (univariate analysis)	2.42∼3.18	1.74∼4.45	0.250
OS (multivariate analysis)	1.74∼2.51	1.18∼4.70	0.152
DSS (univariate analysis)	1.54∼2.05	1.05∼3.04	0.205
DSS (multivariate analysis)	1.43∼3.04	0.88∼6.31	/
DFS (univariate analysis)	2.68∼2.98	1.29∼7.85	/
Recurrence vs. No recurrence	1.79∼3.32	1.21∼7.03	0.465
**CD147 expression in different bladder tissues**			
Bladder cancer vs. Noncancer tissues	40.35∼51.79	20.81∼102.95	0.565
Bladder cancer vs. bladder embryonic tissues	25∼73.51	1.34∼1297.91	/
Bladder cancer vs. Normal bladder tissues	34.71∼49.99	14.50∼127.36	0.023
Bladder cancer vs. Para-carcinoma tissues	52.61∼68.06	8.39∼552.26	/
**CD147 expression with clinicopathological parameters**			
III∼IV stage vs. I∼II stage	73.89∼73.89	4.13∼1322.54	/
pT2-T4 vs. pTa-T1	2.71∼3.52	1.73∼6.01	0.199
Lymph node metastasis vs. No lymph node metastasis	1.65∼3.13	0.53∼8.36	0.653
Low differentiation vs. Moderate & high differentiation	3.68∼5.78	1.62∼14.84	0.193
Male vs. Female	1.25∼1.61	0.80∼2.82	0.225

P < 0.05, exist Publication Bias.

## DISCUSSION

CD147, also known as EMMPRIN, is a transmembrane glycoprotein in the immunoglobulin superfamily. It is highly expressed in many types of malignant tumors, including breast carcinoma, gastrointestinal cancer, prostate cancer, and bladder cancer, and plays a significant role in tumor proliferation, invasion, metastasis, as well as in many other processes in tumorigenesis and tumor development [37–39]. In the current meta-analysis, we pooled 25 datasets from 24 studies [4, 7, 15-24, 25-36] and demonstrated a notable association between CD147 expression and patients with bladder cancer.

According to our results, CD147 expression is closely associated with prognostic and clinicopathological characteristics of bladder cancer. Irrespective of whether the parameter tested was OS, DSS, or DFS, univariate analysis showed that patients with higher CD147 positive expression were more likely to have worse prognosis. Multivariate analysis for OS and DFS gave the same result. However, regarding multivariate analysis for DSS, our results showed no difference between CD147 positive and negative expression groups. Besides this, our results also indicated that CD147 positive expression is correlated with higher rates of recurrence. Our findings relating to survival rate were in accordance with those of other studies comparing CD147 positive and negative expression with OS, DSS, or DFS in breast, ovarian, and gastrointestinal cancers [40–42]. Results of subgroup analysis based on IHC and cut-off values were in accordance with our main results.

Furthermore, CD147 expression is associated with the response to chemotherapy for bladder cancer, and Misra et al. [43] demonstrated that CD147 enhances tumor growth and chemoresistance via the phosphatidylinositol 3-kinase (PI3K)/Akt pathway in a hyaluronan-dependent manner. Als et al. [4] found that the response rates in patients with CD147-negative and CD147-positive tumors were 74% and 39% respectively, with an odds ratio of 4.41 (95% CI=[1.91-10.1]). In addition, by silencing CD147 via RNA interference *In vitro*, Afonso et al. [16] showed a lower cisplatin IC_50_ for bladder cancer (from 24.11 μg/ml to 7.42 μg/ml). That is, the downregulation of CD147 sensitizes bladder cancer cells to chemotherapy.

As CD147 can promote the activity and expression of MCT-1 and MCT-4, forming complexes on the membrane to transport lactic acid produced by anaerobic tumor glycolysis, its expression may be increased in tumor tissues [42, 44]. As such, we investigated CD147 expression in different bladder tissues. The results showed that CD147 expression in bladder cancer tissues is greater than that in non-cancer tissues including embryonic bladder tissues, normal bladder tissues, and para-carcinoma tissues.

Next, we compared the expression of CD147 in relation to recurrence, clinical stages, invasive depth, lymph node metastasis, histological differentiation, and sex, to explore the correlation of CD147 expression with these clinicopathological parameters. In summary, CD147 expression is strongly associated with more advanced clinical stages, greater invasive depth, and poorer histological differentiation. However, CD147 expression is not correlated with lymph node metastasis or sex. As CD147 can facilitate the secretion of matrix metalloproteinase (MMP)-1, MMP-3, MMP-9, and membrane type-1 MMP, it may bring about the degradation of the basement membrane and extracellular matrix. This is one of the major mechanisms of its promotion of tumor metastasis [37, 45]. The meta-analyses of Huang et al. [41] and Peng et al. [46] also indicated that CD147 expression is associated with lymph node metastasis in gastrointestinal and prostate cancers. However, we found no association between CD147 positive expression and lymph node metastasis. However, when we excluded the study of Gao et al. [32], this result was reversed. Therefore, further qualified studies are needed to assess the relationship between CD147 and lymph node metastasis in bladder cancer.

Besides the immunohistochemistry of CD147 protein levels analyzed in this study, other researches are available. Using Western blotting, Xue et al. [15] showed higher CD147 expression in bladder cancer cell lines T24, SCaBER, 5637, BIU-87, and J82 than in the normal urothelial cell line SV-HUC-1. In particular, the T24 and SCaBER cell lines had the highest CD147 protein levels. Bhagirath et al. [26] compared the serum concentration of CD147 in bladder cancer patients and healthy people by enzyme-linked immunosorbent assay and found a significant increase in CD147 levels in the serum samples of bladder cancer patients. Similarly, Li et al. [21] demonstrated higher CD147 mRNA levels in bladder transitional cell carcinoma (59.7%) than in normal bladder tissue (0%), but found no difference with clinical stage (r=0.048, P=0.698) or histological differentiation (r=0.222, P=0.071). Min et al. [22] also found upregulation of CD147 in bladder cancer at both mRNA and protein levels (mRNA: bladder cancer tissues 0.967±0.133, paracarcinoma tissues 0.223±0.096; protein: bladder cancer tissues 0.766±0.103, paracarcinoma tissues 0.165±0.055).

To our knowledge, this meta-analysis is the first to report the relationship between CD147 and bladder cancer. We analyzed 24 high-quality studies (NOS≥7 points) with significant results. Meanwhile, some limitations need to be acknowledged. First, our analysis is based upon published studies found in the literature, but we failed to obtain any unpublished data. Second, patients we included are mostly from China, which limits the universality of our results. Third, different studies used different criteria for CD147 expression, with different follow-up times. This may result in some bias. Nevertheless, we conducted subgroup analyses based on IHC and cut-off values, and this indicates that our results were reliable.

In this meta-analysis, we demonstrated that CD147 expression is increased in bladder cancer tissues compared with non-cancer tissues. This is strongly correlated with poorer OS, DSS, and DFS; recurrence; advanced clinical stages; greater invasive depth; and poorer histological differentiation. However, it was not correlated with lymph node metastasis or sex. In summary, CD147 could be an important diagnostic and prognostic biomarker for bladder cancer.

## MATERIALS AND METHODS

### Search strategy

We searched PubMed (1966-2016), EMBASE (1980-2016), the Cochrane Library (1996-2016), Web of Science (1945-2016), China National Knowledge Infrastructure (1982-2016), and the WanFang databases (1988-2016). The studies were restricted to humans, but not restricted by date, language, or publication status. The following combined search terms were used: (Bladder Neoplasm*, Bladder Tumor*, Bladder Cancer*, Bladder Carcinoma, Bladder Transitional Cell Carcinoma, Bladder Squamous Cell Carcinoma, Bladder Adenocarcinoma) AND (CD147, Extracellular matrix metalloproteinase inducer, EMMPRIN, BSG). We combined the term appropriately with MeSH Terms and used an appropriate adjustment for different databases. Details of the search strategies can be found in Appendix 1.

### Criteria for including studies

1. Published or unpublished case control study or cohort study in English or Chinese with the full text available;
2. All cases had survival data or clinical pathological characteristic data, without radiotherapy or chemotherapy or biological therapy before sampling;
3. Diagnosis of bladder cancer was proven by pathological methods;
4. Studies of CD147 expression based on primary bladder cancer tissue (via either biopsy or surgical), rather than serum or any other kinds of indirect specimen were included;
5. The best quality study was retained for dealing duplicated study.

### Criteria for excluding studies

1. Cell or animal studies, case reports, letters, reviews;
2. The standard of pathological diagnosis was not clear.

### Assessment of included studies

The Newcastle-Ottawa quality assessment scale of case control studies (NOS) [47] was adopted to assess the quality of included studies. It has three categories (selection, comparability, and exposure) and eight items. The quality assessment values ranged from 0 to 9 stars. Studies scored more than 6 stars was included for our analysis.

### Statistical analysis

Literature were independently filtered by two authors to exclude unrelated studies. Then, full texts were independently reviewed, and controversies were solved by discussion. Data were extracted independently by two authors. The software Revman 5.3 and Stata 14.0 were applied. Results were showed with odds ratios (OR), standard mean difference (SMD) or HR (hazard ratio) with 95% confidence intervals (95% CI). Fixed-effects model was adopted for non-heterogeneous data (P > 0.1 and I^2^ < 50%); otherwise, random-effects model was used. If possible, heterogeneity was explored and subgroup analyses were performed.

Additionally, sensitivity analysis was performed to evaluate the influences of individual studies on the final effects size if the parameter has no less than three datasets for parameter. Otherwise, analysis based on different models was used.

Finally, publication bias was assessed by Egger's test (P < 0.05 was considered statistically significant). If publication bias was confirmed, a trim-and-fill method developed by Duval and Tweedie was implemented to adjust for this bias. Then, we replicated the funnel plot with their ‘‘missing’’ counterparts around the adjusted summary estimates [48].

## SUPPLEMENTARY MATERIALS TABLE AND APPENDIX







## Abbreviations

OR: odds ratio
SMD: standard mean difference
HR: hazard ratio
MCT: mono-carboxylate transporter
EMMPRIN: extracellular matrix metalloproteinase inducer
DSS: disease specific survival
IHC: immunohistochemistry
NOS: Newcastle-Ottawa quality assessment scale
OS: overall survival
CI: confidence intervals
DFS: disease recurrence-free survival
SP: streptavidin-perosidase
PI3K: phosphatidylinositol 3-kinase
IC: inhibitory concentration
MMP: matrix metalloproteinase
BSG: basigin
