# Supplementary material for: CD147 as a novel biomarker for predicting the prognosis and clinicopathological features of bladder cancer: a meta-analysis
Source: Oncotarget. 2017 Jul 15;8(37):62573–88. doi: 10.18632/oncotarget.19257 (PMC5617530; doi:10.18632/oncotarget.19257)
Supplement: Supplementary file 1 [file oncotarget-08-62573-s001.pdf]

CD147 as a novel biomarker for predicting the prognosis and clinicopathological features of bladder cancer: a meta-analysis

SUPPLEMENTARY MATERIALS

Supplementary Table 1: Qualitative assessment of included study.

See Supplementary File 1

Appendix 1: Literature searching strategy.

See Supplementary File 2
