# Supplementary material for: CD147 as a novel biomarker for predicting the prognosis and clinicopathological features of bladder cancer: a meta-analysis
Source: Oncotarget. 2017 Jul 15;8(37):62573–88. doi: 10.18632/oncotarget.19257 (PMC5617530; doi:10.18632/oncotarget.19257)
Supplement: Supplementary file 2 [file oncotarget-08-62573-s002.docx]

**Supplementary Table 1: Qualitative assessment of included study**

| Column | Entries | Study | | | | | | | |
| --- | --- | --- | --- | --- | --- | --- | --- | --- | --- |
|  |  | 1 | 2 | 3 | 4 | 5 | 6 | 7 | 8 |
|  | Is the definition adequate | ☆ | ☆ | ☆ | ☆ | ☆ | ☆ | ☆ | ☆ |
|  | Representativeness of the cases | ☆ | ☆ | ☆ | ☆ | ☆ | ☆ | ☆ | ☆ |
| Section | Selection of controls |  |  |  |  |  |  |  |  |
|  | Definition of controls | ☆ | ☆ | ☆ | ☆ | ☆ | ☆ | ☆ | ☆ |
| Comparability | Comparability of cases and controls on the basis of the design and analysis | ☆☆ | ☆ | ☆ | ☆ | ☆ | ☆ | ☆☆ | ☆ |
|  | Ascertainment of exposure | ☆ | ☆ | ☆ | ☆ | ☆ | ☆ | ☆ | ☆ |
| Exposure | Same method of ascertainment for cases and controls | ☆ | ☆ | ☆ | ☆ | ☆ | ☆ | ☆ | ☆ |
|  |  |  |  |  |  |  |  |  |  |
|  | Non-Response rate | ☆ | ☆ | ☆ | ☆ | ☆ | ☆ | ☆ | ☆ |
| Total scores |  | 8 | 7 | 7 | 7 | 7 | 7 | 8 | 7 |

1. Afonso, J 2015 [16]; 2. Hemdan, T 2015 [17]; 3. Li, F 2015 [25]; 4. Choi, JW 2014 [7]; 5. Min, L 2014 [22]; 6. El-Rehim, DM 2013 [20]; 7. Bhagirath, D 2012 [26]; 8. Wittschieber, D 2011 [27].

| Column | Entries | Study | | | | | | | |
| --- | --- | --- | --- | --- | --- | --- | --- | --- | --- |
|  |  | 9 | 10 | 11 | 12 | 13 | 14 | 15 | 16 |
|  | Is the definition adequate | ☆ | ☆ | ☆ | ☆ | ☆ | ☆ | ☆ | ☆ |
|  | Representativeness of the cases | ☆ | ☆ | ☆ | ☆ | ☆ | ☆ | ☆ | ☆ |
| Section | Selection of controls |  |  |  |  |  |  |  |  |
|  | Definition of controls | ☆ | ☆ | ☆ | ☆ | ☆ | ☆ | ☆ | ☆ |
| Comparability | Comparability of cases and controls on the basis of the design and analysis | ☆☆ | ☆ | ☆ | ☆☆ | ☆☆ | ☆ | ☆ | ☆ |
|  | Ascertainment of exposure | ☆ | ☆ | ☆ | ☆ | ☆ | ☆ | ☆ | ☆ |
| Exposure | Same method of ascertainment for cases and controls | ☆ | ☆ | ☆ | ☆ | ☆ | ☆ | ☆ | ☆ |
|  |  |  |  |  |  |  |  |  |  |
|  | Non-Response rate | ☆ | ☆ | ☆ | ☆ | ☆ | ☆ | ☆ | ☆ |
| Total scores |  | 8 | 7 | 7 | 8 | 8 | 7 | 7 | 7 |

9. Xue, YJ 2011 [15]; 10. Gao, LJ 2011 [24]; 11. Li, M 2011 [21]; 12. Han, ZD 2010 [19]; 13. Zhong, WD 2010 [18]; 14. Chen, QB 2010 [28]; 15. Cui, W 2010 [29]; 16. He, HC 2009 [30].

| Column | Entries | Study | | | | | | | |
| --- | --- | --- | --- | --- | --- | --- | --- | --- | --- |
|  |  | 17 | 18 | 19 | 20 | 21 | 22 | 23 | 24 |
|  | Is the definition adequate | ☆ | ☆ | ☆ | ☆ | ☆ | ☆ | ☆ | ☆ |
|  | Representativeness of the cases | ☆ | ☆ | ☆ | ☆ | ☆ | ☆ | ☆ | ☆ |
| Section | Selection of controls |  |  |  |  |  |  |  |  |
|  | Definition of controls | ☆ | ☆ | ☆ | ☆ | ☆ | ☆ | ☆ | ☆ |
| Comparability | Comparability of cases and controls on the basis of the design and analysis | ☆ | ☆ | ☆☆ | ☆ | ☆ | ☆ | ☆ | ☆ |
|  | Ascertainment of exposure | ☆ | ☆ | ☆ | ☆ | ☆ | ☆ | ☆ | ☆ |
| Exposure | Same method of ascertainment for cases and controls | ☆ | ☆ | ☆ | ☆ | ☆ | ☆ | ☆ | ☆ |
|  |  |  |  |  |  |  |  |  |  |
|  | Non-Response rate | ☆ | ☆ | ☆ | ☆ | ☆ | ☆ | ☆ | ☆ |
| Total scores |  | 7 | 7 | 8 | 7 | 7 | 7 | 7 | 7 |

17. Peng, XH 2009 [23]; 18. Lin, JX 2008 [31]; 19. Als, AB 2007 [4]; 20. Gao, L 2007 [32]; 21. Li, M 2007 [33]; 22. Li, WL 2007 [34]; 23. Han, JL 2003 [35]; 24. Muraoka, K 1993 [36].
