# Supplementary material for: CD147 as a novel biomarker for predicting the prognosis and clinicopathological features of bladder cancer: a meta-analysis
Source: Oncotarget. 2017 Jul 15;8(37):62573–88. doi: 10.18632/oncotarget.19257 (PMC5617530; doi:10.18632/oncotarget.19257)
Supplement: Supplementary file 3 [file oncotarget-08-62573-s003.docx]

1. Pubmed

n=25

(Urinary Bladder Neoplasms[MeSH Terms] OR (Bladder[Title/Abstract] AND (Neoplasm*[Title/Abstract] OR Tumor*[Title/Abstract] OR Cancer*[Title/Abstract] OR Carcinoma[Title/Abstract]))) AND (Antigens, CD147[MeSH Terms] OR CD147[Title/Abstract] OR (Extracellular[Title/Abstract] AND matrix[Title/Abstract] AND metalloproteinase[Title/Abstract] AND inducer[Title/Abstract]) OR EMMPRIN[Title/Abstract] OR BSG[Title/Abstract])

1. EMBASE

n=41

('bladder cancer'/exp OR (Bladder:ti,ab AND (Neoplasm*:ti,ab OR Tumor*:ti,ab OR Cancer*:ti,ab OR Carcinoma:ti,ab))) AND ('CD147 antigen'/exp OR CD147:ti,ab OR (Extracellular:ti,ab AND matrix:ti,ab AND metalloproteinase:ti,ab AND inducer:ti,ab) OR EMMPRIN:ti,ab OR BSG:ti,ab) AND [embase]/lim

1. Cochrane Library

n=1

([mh "Urinary Bladder Neoplasms"] OR (Bladder:ti,ab AND (Neoplasm*:ti,ab OR Tumor*:ti,ab OR Cancer*:ti,ab OR Carcinoma:ti,ab))) AND ([mh "Antigens, CD147"] OR CD147:ti,ab OR (Extracellular:ti,ab AND matrix:ti,ab AND metalloproteinase:ti,ab AND inducer:ti,ab) OR EMMPRIN:ti,ab OR BSG:ti,ab)

1. Web of Science

n=26

(TS=Bladder AND (TS=Neoplasm* OR TS=Tumor* OR TS=Cancer* OR TS=Carcinoma)) AND (TS=CD147 OR (TS=Extracellular AND TS=matrix AND TS=metalloproteinase AND TS=inducer) OR TS=EMMPRIN OR TS=BSG)

1. China National Knowledge Infrastructure

n=29

(SU='膀胱肿瘤' OR SU='膀胱癌') AND (SU='CD147' OR SU='抗原, CD147' OR SU='细胞外基质金属蛋白酶诱导因子')

Note: this database is Chinese-based so we translate the search terms into Chinese.

1. WanFang Database

n=25

(主题:(膀胱肿瘤) + 主题:(膀胱癌)) * (主题:(CD147) + 主题:(抗原, CD147) + 主题:(细胞外基质金属蛋白酶诱导因子))

Note: this database is Chinese-based so we translate the search terms into Chinese.
